# Microbial cell wall sorption and Fe–Mn binding in rhizosphere contribute to the obstruction of cadmium from soil to rice

**DOI:** 10.3389/fmicb.2023.1162119

**Published:** 2023-04-17

**Authors:** Jie Li, Yi-Kai Guo, Qing-Xia Zhao, Ji-Zheng He, Qian Zhang, Hong-Ying Cao, Chao-Qiong Liang

**Affiliations:** ^1^State Key Laboratory of Mycology, Institute of Microbiology, Chinese Academy of Sciences, Beijing, China; ^2^Ecological Environment Planning and Environmental Protection Technology Center of Qinghai Province, Xining, China; ^3^Institute of New Rural Development, Guizhou University, Guiyang, China; ^4^Faculty of Veterinary and Agricultural Sciences, The University of Melbourne, Parkville, VIC, Australia; ^5^Key Laboratory of Land Surface Pattern and Simulation, Beijing Key Laboratory of Environmental Damage Assessment and Remediation, Institute of Geographic Sciences and Natural Resources Research, Chinese Academy of Sciences, Beijing, China; ^6^Shaanxi Academy of Forestry, Xi'an, China

**Keywords:** cadmium, bio-sorption, tolerant species, Cd accumulation, rice

## Abstract

Screening high-tolerant microorganisms to cadmium (Cd) and revealing their bio-obstruction mechanism could be significant for Cd regulation from farmland to the food chain. We examined the tolerance and bio-removal efficiency of Cd ions of two bacterial strains, *Pseudomonas putida* 23483 and *Bacillus sp*. GY16, and measured the accumulation of Cd ions in rice tissues and its different chemical forms in soil. The results showed that the two strains had high tolerance to Cd, but the removal efficiency was decreased successively with increasing Cd concentrations (0.05 to 5 mg kg^−1^). Cell-sorption accounted for the major proportion of Cd removal compared with excreta binding in both strains, which was conformed to the pseudo-second-order kinetics. At the subcellular level, Cd was mostly taken up by the cell mantle and cell wall, and only a small amount entered into the cytomembrane and cytoplasmic with time progressed (0 to 24 h) in each concentration. The cell mantle and cell wall sorption decreased with increasing Cd concentration, especially in the cytomembrane and cytoplasmic. The scanning electron microscope (SEM) and energy dispersive X-ray (EDS) analysis verified that Cd ions were attached to the cell surface, and the functional groups of C-H, C-N, C=O, N-H, and O-H in the cell surface may participate in cell-sorption process tested by the FTIR analysis. Furthermore, inoculation of the two strains significantly decreased Cd accumulation in rice straw and grain but increased in the root, increased Cd enrichment ratio in root from soil, decreased Cd translocation ratio from root to straw and grain, and increased the Cd concentrations of Fe–Mn binding form and residual form in rhizosphere soil. This study highlights that the two strains mainly removed Cd ions in solution through biosorption and passivated soil Cd as Fe–Mn combined form ascribe to its characteristics of manganese-oxidizing, eventually achieving bio-obstruction of Cd from soil to rice grain.

## 1. Introduction

Cadmium (Cd) resulting from industrial and mining activities can bring health risks such as liver injury or osteoporosis through the food chain (Jin et al., 2020; Peng et al., 2021). Conventional physical and chemical techniques for Cd contamination remediation may bring secondary pollution to soil structure and organisms (Li et al., 2017; Mukherjee et al., 2022). For example, physicochemical techniques with specialized equipment are often accompanied by a broken of soil properties and structures, and bring secondary pollution to soil fauna and microorganisms in farmland (Beleza et al., 2001). Thus, eco-friendly techniques of bioremediation are urgently needed in field remediation to mitigate the Cd bioavailability, and consequently to reduce Cd accumulation in food. As a low-cost and environmentally friendly approach, micro-remediation was advocated in practice by using resistant and functional microorganisms (Alluri et al., 2007; Su et al., 2020). In polluted farmland, Cd could be immobilized by highly tolerant microorganisms through bioaccumulation and precipitation, *via* exogenous addition, which could help in reducing its accumulation in crops (Farhadian et al., 2008; Mengual et al., 2016; Peng et al., 2021). Therefore, screening for highly tolerant and efficient microorganisms in Cd immobilization could be essential for Cd regulation from farmland to the food chain.

For toxic heavy metals, physisorption (Pabst et al., 2010), complexation, and chelation of microbial cells (Shim et al., 2015) are the primary micro-actions. Therefore, the exchanging of positive ions such as Ca^2+^, Mg^2+^, and H^+^ on the cell surface and the combining ability of bio-macromolecules are responsible for the efficiency of microbial cell adsorption (Wu et al., 2009; Peraferrer et al., 2012; Rukshana et al., 2012), while the active groups such as -SO_3_H, -COOH, and -NH in the cell surface participate in chelation (Blanco, 2000; Peraferrer et al., 2012). In addition, inorganic deposition such as phosphorus salt, sulfate, carbonate, and hydroxide formed with Cd in cells (Özdemir et al., 2009; Jafar Mazumder, 2020), and complex precipitated compounds induced by microbial metabolites (Mutiat et al., 2018; Jafar Mazumder, 2020) are efficient microbial passivating forms to reduce the bioactivity of Cd. Furthermore, for many highly tolerant microorganisms, Cd could be fixed inside the cells without causing serious damage or pumped out after transformation depending on various transport systems (Shim et al., 2015; Li et al., 2019). However, bio-removal efficiency and mechanisms of Cd by microbial species remain unclear (Sulaymon et al., 2013; Yin et al., 2019).

*In situ* immobilization of Cd^2+^ by efficient microbes is considerable in practice because of less effect on agricultural planning (Shi et al., 2021). High-tolerant microbes could mediate the mobility and availability of Cd by releasing chelating agents, redox changes, and phosphate solubilization (Idris et al., 2004; Manoj et al., 2020). For example, resistant microbes participated in solid-solution partitioning processes of soil Cd *via* absorption–desorption by creating organic compounds such as organic acids, biosurfactants, and siderophores, as well as oxidation and reduction reactions (Braud et al., 2009; Arwidsson et al., 2010; Venkatesh and Vedaraman, 2012). Moreover, in natural soil, manganese (Mn) oxides have a high ability to adsorb Cd ions due to their large negative charge, and Cd could even enter into the structure of Mn oxide to be fixed (Muller et al., 2002). Nature Mn oxidation often proceeds slowly (Nealson et al., 1988), while the rate could be accelerated when microorganisms are involved, consequently increasing the heavy metal adsorption of Mn oxides (Kim et al., 2003; Meng et al., 2009). Some functional microbes such as *Pseudomonas putida* MnB1, *Leptothrix discophora* SS-1 (Caspi et al., 1996), and *Bacillus sp. SG-1* (Van Waasbergen et al., 1996) are said to participate in the Mn oxidation process in various ways (Villalobos et al., 2003; Saratovsky et al., 2006) and to synthesize high-activity biogenic nano-sized Mn oxides (Kim et al., 2003). Those biogenic Mn oxides are assumed to have high adsorption efficiency and sensitive reaction with heavy metal ions compared with nature ones (Sasaki et al., 2008; Meng et al., 2009). A previous study showed that the Cd maximum adsorption ability of biogenic Mn oxides is significantly stronger than natural todorokite (Meng et al., 2009). Therefore, the interactions among Cd, biogenic Mn oxides, and Cd-tolerant microbes could be potentially highly efficient for bioremediators to adsorb, degrade, convert, and neutralize Cd as a bioremediation method in Cd-contaminated environments. However, there is seldom research on bioremediation efficiency and mechanism of Cd using Mn oxide microbes in the soil.

Rice (*Oryza sativa*) is one of the world's most important crops (Hu et al., 2016; Negrini et al., 2020) with the strongest ability of Cd enrichment and absorption (Suksabye et al., 2016; Negrini et al., 2020). “Cadmium rice” has become the main source of Cd intake for rice-eating people in East Asia such as China and Japan (Tsukahara et al., 2003; Honma et al., 2016). In this study, two bacterial strains, *Pseudomonas putida* 23483 and *Bacillus* sp. GY16 with functions of Mn oxidation (Meng et al., 2009), were used for revealing the biosorption mechanism of Cd, and the bio-obstruction of Cd accumulation on rice plants, and its bio-immobilization in soil. The bio-removal pattern and subcellular accumulation of Cd were investigated under a gradient concentration. The scanning electron microscope (SEM) and the energy dispersive X-ray (EDS) spectra were used to test microbial cell surface sorption of Cd, and the Fourier transform infrared (FTIR) spectra were used to verify the functional groups that participated in cell-sorption. Eventually, the Cd accumulation on rice plant tissues and changes in chemical forms of Cd in soil were measured to reveal the bio-obstruction efficiency of the two strains based on a pot control experiment. We hope to provide theoretical guidance for field application and improve the bioremediation efficiencies under *in situ* conditions.

## 2. Experimental section

### 2.1. Preparation of bacterial strains

Microbial strains, *Pseudomonas putida* 23483 (*P. putida*) and *Bacillus sp*. GY16, were selected as the target microbial species, purchased from China General Microbiological Culture Collection Center (CGMCC), and isolated from Fe–Mn nodules from Hubei Province, Central China, respectively (He et al., 2008). The strains were cultured in LB medium (tryptone 10 g, yeast extract 5 g, NaCl per liter, 5 g, pH = 7.0).

### 2.2. Tolerance test of the two strains

To test Cd tolerance of the two strains, the growth performance was determined in LB liquid medium under Cd concentration gradients of 0, 0.05, 0.5, 5, 10, 50, and 100 mg L^−1^. The strains were incubated at 28°C for 72 h in 150-ml Erlenmeyer flasks, at 180 rpm using a thermostat shaker. The absorbance value of the solution was measured at 600 nm at the time point of 0, 6, 12, 24, 36, 48, 60, and 72 h using a microplate reader (BioTek Synergy H1), and the relative growth was calculated by the ratio of absorbance in each time point to that in point 0 h.

### 2.3. Removal efficiency of the strains

Based on the Cd tolerance test, the biosorption experiment was arranged under Cd concentrations of 0.05, 0.5, and 5 mg L^−1^, and under this gradient, the strains were not cruelly restrained but grown well. First, the strains were cultured in the LB liquid medium for 24 h until the late exponential phase, and the cells were isolated after centrifugation at 6000 *g* for 10 min and then the adhered media were washed out with sterilized 0.05 M KNO_3_ solution (pH 7.0) three times. Second, we resuspended the cells in 0.05 M KNO_3_ solution (Huang et al., 2005) in three 150-ml triangular flasks with a biomass concentration of 8 × 10^9^ cells ml^−1^, and adjusted Cd to three concentrations as 0.05, 0.5, and 5 mg L^−1^ by adding CdCl_2_ (Cd concentrations of 0.05, 0.5, and 5 mg L^−1^ in KNO_3_ solution without microbial cells as control). Third, the reaction solution of each concentration was sub-packaged into 2-ml tubes, with six replicates, and was placed in a thermostatted shaker at the conditions of 37°C, 180 rpm. We collected the solution at 0.5, 1, 1.5, 2.5, 3.5, 6, 12, and 24 h time nodes, the solution was centrifuged at 12,000 *g* for 5 min, and then, the supernatant was collected in clean tubes for Cd measurement (Microbial removal Cd). Finally, the leaving substrate precipitations were digested with 2 ml of concentrated nitric acid for 1 h (cell-sorption Cd) and analyzed for Cd concentrations by coupled plasma-mass spectrometry (ICP-MS). The excreta binding amount (excreta binding Cd) of the two microbial species = the amount of microbial removal Cd – the amount of cell-sorption of Cd.

### 2.4. Subcellular fractionation of the strains

The subcellular fractionation was isolated step by step according to the method of Kumar and Upretil (2000). First, the solution samples (six replicates) collected at each time node mentioned before were centrifuged for 5 min at 12,000 *g*, and the supernatant was discarded but the substrate precipitations were maintained. Then, the precipitations were washed three times with Tris buffer (1 mL, 0.03 mol L^−1^) containing 0.1 mol L^−1^ EDTA, adjusted pH to 8.0, and centrifuged three times at 12,000 *g* for 2 min. The centrifuged Tris buffer was collected together in clean tubes and the substrate precipitations were maintained. Second, the substrate precipitations were resuspended in the Tris buffer. We added lysozyme and adjusted it to 400 μg ml^−1^ with the precipitations, and incubated it at 25°C for 30 min to prepare spheroplasts. When finished, the spheroplasts were centrifuged at 2,576 *g* for 15 min under 0–4°C; the supernatant Tris buffer was a periplasmic fluid that consisted of a peptidoglycan layer and was obtained in clean tubes. The precipitations (spheroplasts) were resuspended in Tris buffer (pH = 8.1). Third, the spheroplasts were disrupted completely with the ultrasonic cell crusher by two 30 s bursts and then centrifuged at 12,000 *g* for 2 min to remove debris. The supernatants (cytoplasm) were collected into new test tubes, and the precipitations (crude membrane) consisting of both outer and inner membrane envelopes were maintained. Finally, the supernatants and the crude membrane in tubes were digested by concentrated nitric acid (2 ml) at 60°C for 1 h and analyzed for Cd concentrations by ICP-MS.

### 2.5. SEM, EDX, and FTIR spectra analysis

The strains were cultured in the LB medium at 28°C with Cd ions of 50 mg kg^−1^ and without Cd ions for 48 h. The medium was washed clearly with deionized water, and the microbial cells were resuspended and immobilized in 0.05 M KNO_3_ solution containing 2.5% glutaraldehyde for 4 h. When finished, the solution was centrifuged at 8,000 g for 5 min and maintained the precipitations, washed three times with 0.05 M KNO_3_ solution, and centrifuged at 8,000 *g* for 5 min. The precipitations were maintained and were ethanol gradient dehydrated in a sequence of 30, 50, 70, 85, and 90% once, and in 100% ethanol twice, 15 min per time, and centrifuged at 8,000 *g* for 5 min, and the supernatants were removed each time. The precipitations were immersed in a solution of isopentyl acetate twice to replace ethanol at 20 min per time. Then, the precipitations were gradiently frozen at −20°C (for 12 h), −40°C (for 12 h), and −80°C (for 24 h) step by step, and then freeze-dried at −40°C in a freeze drier for 24 h. Finally, the microbial cell powder was placed on SEM and EDS to observe the phenotypic changes and surface ion content, placed on an FTIR instrument (Bruker Tensor 27, Ettlingen, Germany) for *in situ* functional group analysis, and calculated the area and the height of the peaks using OPUS v5.5 software in FTIR analysis.

### 2.6. Greenhouse experiment arrangement

The soil was collected for rice plant cultivation from a Cd-contaminated area (E113°10′ 54″, N 27°30′ 45″), with a depth of 0–30 cm in Sima town, located in LiuYang county, Hunan Province. The air-dried soil was passed through a 2-mm sieve, mixed uniformly, and sterilized using ultraviolet. Soil total Cd concentration was 1.09 mg kg^−1^, available Cd concentration was 0.35 mg kg^−1^, cation exchange capacity was 12.16 cmol kg^−1^ (+), organic matter content was 50.44 g kg^−1^, the total carbon content was 2.6 %, the total nitrogen content was 0.3%, and the C and N ratio was 8.81. For pot experiments in the greenhouse, 3 kg of soil was poured into customized polyethylene plastic square pots with 40 cm height, 30 cm length, and 25 cm width. We arranged two treatments and six replicates for each treatment, including the bacterial inoculum of *P. putida* with 10 mM MnCl_2_ solution (*P. putida*) and the bacterial inoculum of *Bacillus sp*. GY16 with 10 mM MnCl_2_ solution (*Bacillus sp*. GY16). We also arranged soil with 10 mM MnCl_2_ solution (MnCl_2_) and without MnCl_2_ solution as control (control). The two bacterial strains were cultured in LB medium for 48 h and centrifuged to remove the adhered medium, washed, and dispersed in deionized water. Each single inoculum strain contains an amount of 5 × 10^8^ colony-forming units ([cfu] ml^−1^). Afterward, rice seedlings (variety is Zhongjiazao 17) were germinated in sterilization vermiculite and transplanted into pots until grown to a 5 cm height, and one pot received two similar size seedlings. The pots with seedlings were placed in the greenhouse with 15–28°C and 16/8-h of light/dark period, and waterlogging growth was kept for 3 months.

After maturity, watering was stopped and lasted for 1 week, plants were harvested, and soil samples were collected. Roots, straw, and grain were separately oven-dried at 65°C for 72 h, and the biomass was weighed. According to Tessier et al. (1979) and Gleyzes et al. (2002), the fractional extraction method was adopted to extract and separate different forms of Cd in soil samples with a soil–water ratio of 1:2.5 (W/V), including exchangeable Cd (extracted by 1 M MgCl_2_, pH = 7.0), carbonate binding Cd (extracted by 1 M CH_3_COONa, pH = 5.0), iron manganese oxide binding Cd (extracted by 0.04 M NH_2_OH-HCl and 25% CH_3_COOH), organic binding Cd (extracted by 0.02 M HNO_3_ and 30% H_2_O_2_, 85 ± 3°C), and residue Cd (digested by HF-HClO_4_). The plant materials were ground into powder and digested with HNO_3_-HClO_4_ for Cd extraction (Wang et al., 2017). Eventually, the earlier processed samples were analyzed by ICP-MS for Cd concentrations in soil and plant tissues. We also calculated the translocation ratio and the enrichment ratio of Cd to trace that from soil to plant tissues. Taking the trace of soil Cd to plant root, for example, the translocation ratio was calculated by Cd accumulation in root/Cd accumulation in soil, while the enrichment ratio was calculated by Cd accumulation (in root–in soil)/soil Cd in control pots (Jankong and Visoottiviseth, 2008; Chen et al., 2016).

### 2.7. Statistical analysis

The statistical analyses were performed in R version 4.1.3. A one-way ANOVA was used to explore the difference in biosorption capacities among different treatments, and significant differences among treatments were compared using Tukey's honestly significant difference (HSD) test at *p* < 0.05, after the log transformation as the data did not satisfy the normality of distribution or homogeneity of variance.

## 3. Results and discussion

### 3.1. Cd tolerance and removal efficiency

**Figure 1:**
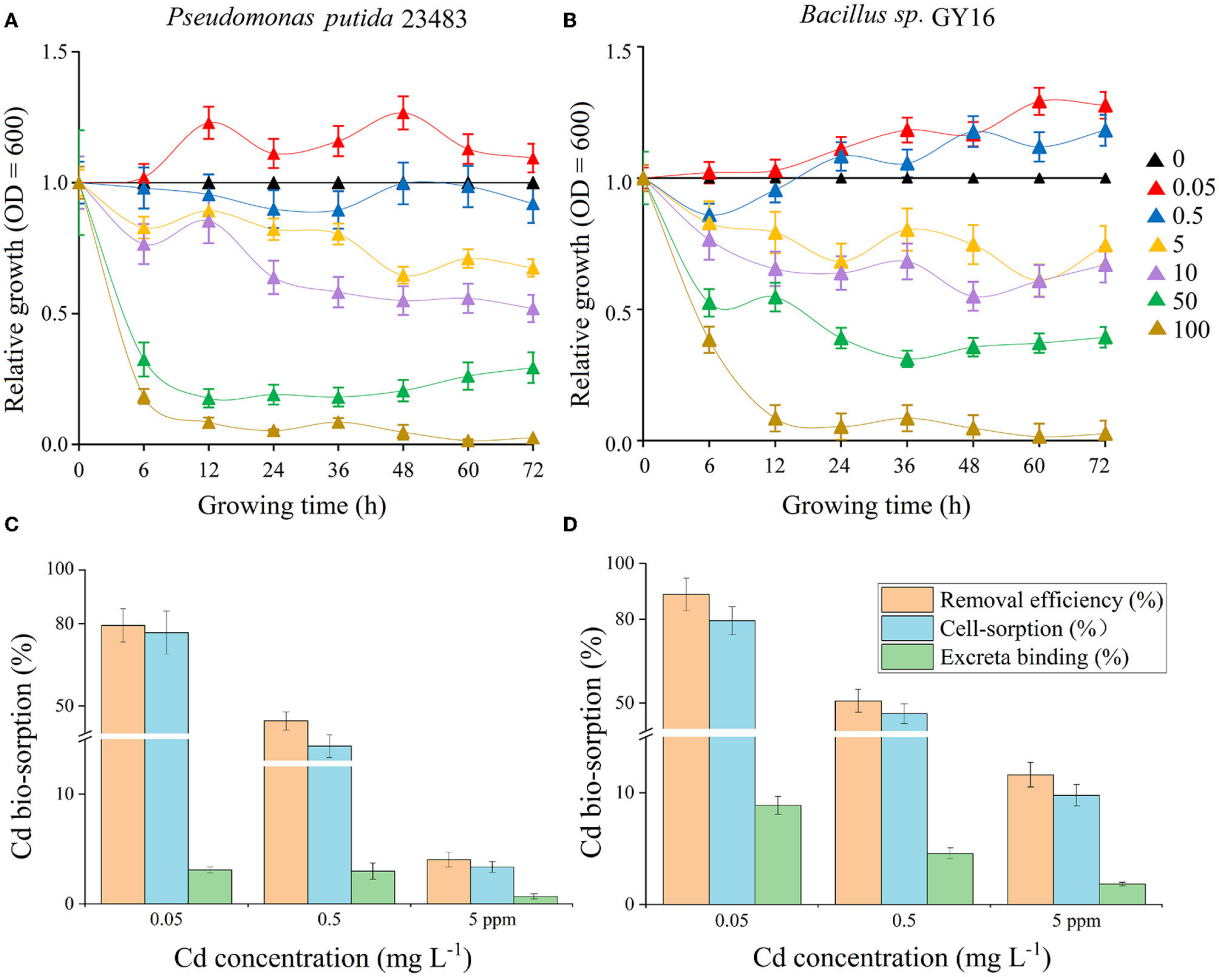
**(A, B)** Tolerance test of Cd concentrations of 0, 0.05, 0.5, 5, 10, 50, and 100 mg L^−1^ on the growth of microbial strains *Pseudomonas putida* 23483 **(A)** and *Bacillus sp*. GY16 **(B)**. The relative growth indicates the growth efficiency in the presence of Cd of different concentrations (0.05–100 mg L^−1^) compared with the growth in the medium without Cd (0 mg L^−1^). **(C, D)** The removal efficiency, cell-sorption, and excreta binding of Cd during the adsorption equilibrium phase (24 h) by *Pseudomonas putida* 23483 **(C)** and *Bacillus sp*. GY16 **(D)** under different Cd concentrations (0.05, 0.5, and 5 mg L^−1^).

Low concentration (0.05–0.5 mg kg^−1^) of Cd was not restrained even promoted microbial growth, and the two strains showed high tolerance to Cd in medium concentrations (5–10 mg kg^−1^), whereas high concentrations (50–100 mg kg^−1^) significantly inhabited the microbial growth (Figures 1A, B). This suggested that the two strains could be Cd-resistant microbes and good candidates for bioremediation, because the concentration of soil total Cd is 1.09 mg kg^−1^ and the available concentration is 0.35 mg kg^−1^ in nature farmland in our study sites. Therefore, we performed a biosorption experiment under the concentrations of 0.05, 0.5, and 5 mg kg^−1^, which, respectively, represented low, medium, and high levels of Cd stress. In line with the previous studies (Bai et al., 2008; Panwichian et al., 2010), we found that the removal efficiency was decreased successively with increasing Cd concentration in each strain (Figures 1C, D). This may be because of lacking sufficient-free sites on the cell surface to combine for more Cd ions (Foo and Hameed, 2010; Chakravarty and Banerjee, 2012), and the increased cell toxicity due to increased Cd concentration (Shafqat et al., 2008; Yu and Fein, 2015). Furthermore, the removal efficiency differs in *P. putida* (79.5–6.9%) and *Bacillus sp*. GY16 (90.3–18.8%), depending on different cellular structures and functions of different microbes (Guo et al., 2010; Shim et al., 2015). We also found that the cell-sorption responsible for Cd removed more than excreta binding in both strains (Figures 1C, D), suggesting the importance of cell wall structures and chemical composition in bio-adsorption (Peraferrer et al., 2012; Rukshana et al., 2012), and the cell-sorption pattern was conformed to the pseudo-second-order kinetics (Table 1). The cell-sorption efficiency increased rapidly in the first 30 min, which may result from rapid binding *via* electrostatic adsorption of metal ligands or *via* complex action of various polar functional groups that existed on the cell wall surface, rather than internalized in the microbial cells (Wu et al., 2009; Ali et al., 2022). After 30/60 min, it reached a steady state for the maximum capacity of biosorption. This may be ascribed to the active transport process of Cd accompanied by energy consumption through microbial metabolism, which was slower than cell wall binding (Bai et al., 2008). It should be pointed out that the experiment was performed in simplex KNO_3_ solution without supplementary nutrients, the microbial secretions were relatively limited, and their reactions of ions or compounds to immobilize Cd was lacking. Therefore, compared with soil conditions, the Cd removal efficiency by microbial-secreted extracellular precipitation may be somehow diminished (Achal et al., 2009).

**Table 1:** Biosorption kinetic models for Cd of *Pseudomonas putida* 23483 and *Bacillus sp*. GY16 under different concentrations of Cd ions.

**Species**	**Cd (mg mL^−1^)**	**Pseudo-first-order kinetics**			**Pseudo-second-order kinetics**		
		**k**_1_ **/min**	**q**_e_ **/mg kg** ^−1^	**R** ^2^	**k**_2_**/ kg mg** ^−1^**min**^−1^	**q**_e_ **/mg kg** ^−1^	**R** ^2^
*Pseudomonas putida* 23483	0.05	0.008	0.003	0.785	9.811	0.049	0.999
	0.5	0.001	0.041	0.660	0.321	0.255	0.999
	5	0.006	0.147	0.944	0.087	0.379	0.999
*Bacillus sp*. GY16	0.05	0.004	0.005	0.860	2.874	0.030	0.999
	0.5	0.005	0.020	0.875	0.271	0.266	0.999
	5	0.004	0.139	0.944	0.080	0.671	0.999

### 3.2. Subcellular sorption of Cd by the strains

**Figure 2:**
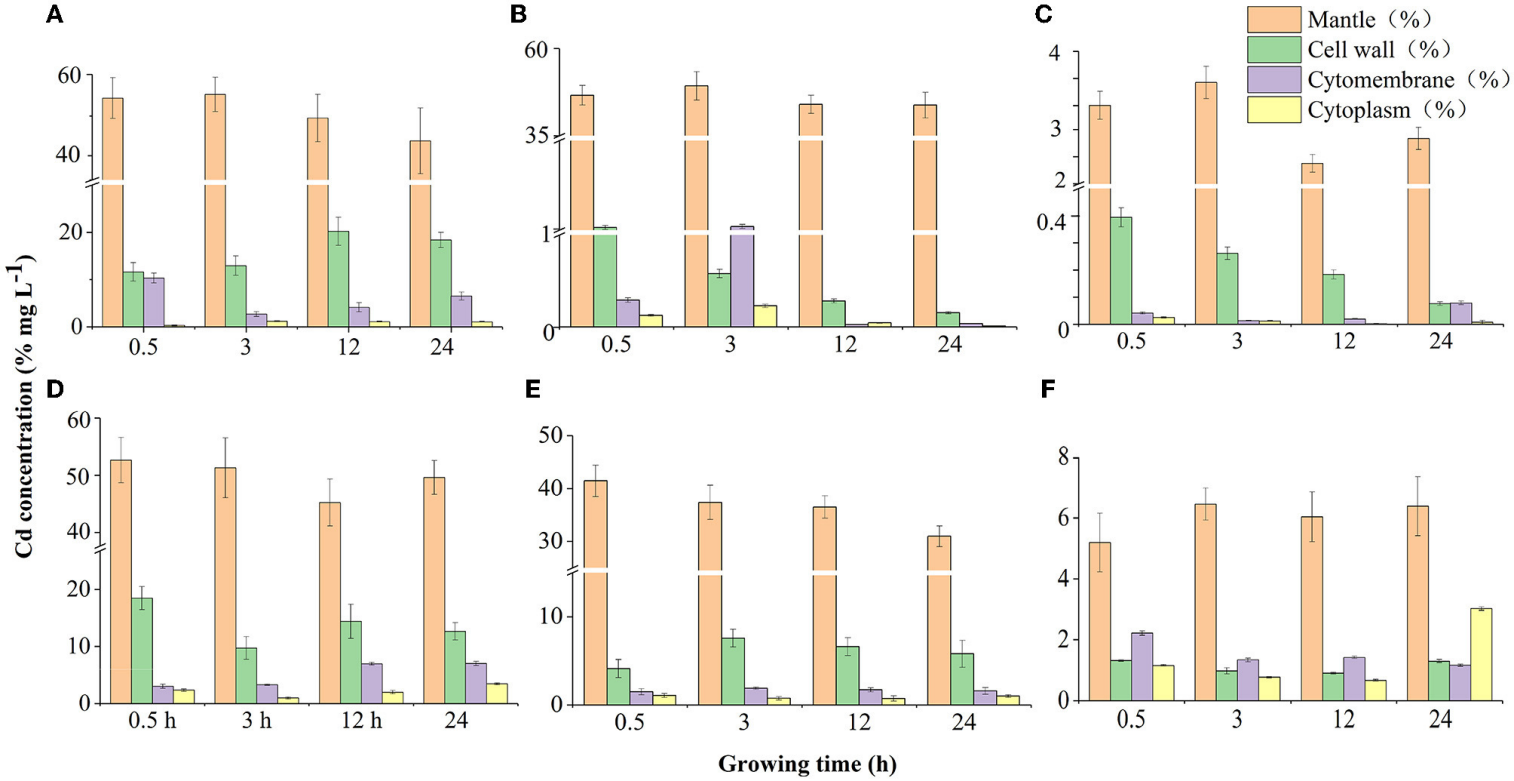
Cd distribution on substructures (cell mantle, cell wall, cytomembrane, and cytoplasm) of two microbial strains in different Cd concentrations during sorption time (0.5, 3, 12, and 24 h). **(A–C)** Cd sorption by *Pseudomonas putida* 23483 in concentrations of 0.05, 0.5, and 5 mg L^−1^, respectively. **(D–F)** Cd sorption by *Bacillus sp*.GY16 in concentrations of 0.05, 0.5, and 5 mg L^−1^, respectively.

We measured Cd concentration in different subcellular fractionations including cell mantle, cell wall, cell cytomembrane, and cytoplasm to ascertain the sorption mechanism of microbial cells. The results showed that Cd accumulation on subcellular fractionations was decreased with increasing concentration (Figure 2). Cd was mainly taken up by microbial cell mantle with 55.2–45.1% (Cd = 0.05 mg mL^−1^), 48.8–40.7% (Cd = 0.5 mg mL^−1^), and 3.7–2.4% (Cd = 5 mg mL^−1^) in *P. putida* (Figures 2A–C) and 53.6–45.2% (Cd = 0.05 mg mL^−1^), 42.1–33.6% (Cd = 0.5 mg mL^−1^), and 6.3–5.2% (Cd = 5 mg mL^−1^) in *Bacillus sp*. GY16 at different concentrations within 24 h (Figures 2D–F). This was in line with previous studies which suggested that most of the Cd was taken up by a cell wall (Kumar and Upretil, 2000; Bai et al., 2008; Ali et al., 2022). Furthermore, the Cd resistance and fixation patterns were adjusted by microorganisms in different Cd stress degrees. For example, at low concentration (0.05 mg L^−1^), Cd was mainly allocated in the cell wall system but barely entered into the cytomembrane (< 3%) and cytoplasmic (< 1%), but less allocated even in the cell wall (< 8%) at the higher concentrations of 0.5 and 5 mg L^−1^. This may be a self-protected mechanism to avoid high concentrations of Cd ions from entering cells and poisoning microbial membrane systems (Zouboulis et al., 2004; Yin et al., 2019).

Moreover, Cd in microbial subcellular fractionations fluctuated as time progressed (Figure 2). At the beginning, Cd ions adhered to the cell mantle ascribed to the cell-intrinsic surface properties, a few were transmitted to cytomembrane cross-inner cell wall with contact time passed, and some even entered into and were stored in cytoplasmic of the cells under low concentration (0.05 mg mL^−1^, Figure 2A). This may be an active transportation accompanied by water/nutrient uptake processes for barely poisoning in low Cd concentration (Marques et al., 2009), and depended on different import mechanisms of sorption, diffusion, complexation, chelation, and micro-precipitation (Franke et al., 2003; Srivastava and Gupta, 2021; Verma et al., 2021). In medium (0.5 mg kg^−1^) and high (5 mg L^−1^) concentrations, as contact time progresses, Cd ions in the inner cell wall first access into cytomembrane, resulting in an increase of Cd accumulated in the cytomembrane (earlier 0.5–3 h). Then, Cd ions in the cytomembrane and cytoplasmic were flowed out and nearly without retention. However, the cell mantle kept the most amount of Cd during the contact time for *P. putida* (Figures 2B, C). The export mechanisms such as CDF race efflux system (Blanco, 2000), CBA, and P type-ATP enzyme (Leedjärv et al., 2008) may play the leading role in this process (Perron et al., 2004; Srivastava and Gupta, 2021; Verma et al., 2021), yet the precise mechanism remains to be further studied. In comparison, Cd ions were stably stocked in the cell mantle, cell wall, cytomembrane, and cytoplasmic of *Bacillus sp*. GY16 with an extension of the contact time in the medium and high concentration (Figures 2E, F), confirmed its high Cd tolerance and import mechanisms (Franke et al., 2003). Therefore, our study confirmed that Cd was not only adsorbed in microbial cell surface (Pabst et al., 2010) but also inside the cells (Shim et al., 2015) *via* two biochemical stages including microbial biosorption and microbial bioaccumulation (Sprocati et al., 2006; Verma et al., 2021).

### 3.3. SEM, EDX, and FTIR spectra analysis

**Figure 3:**
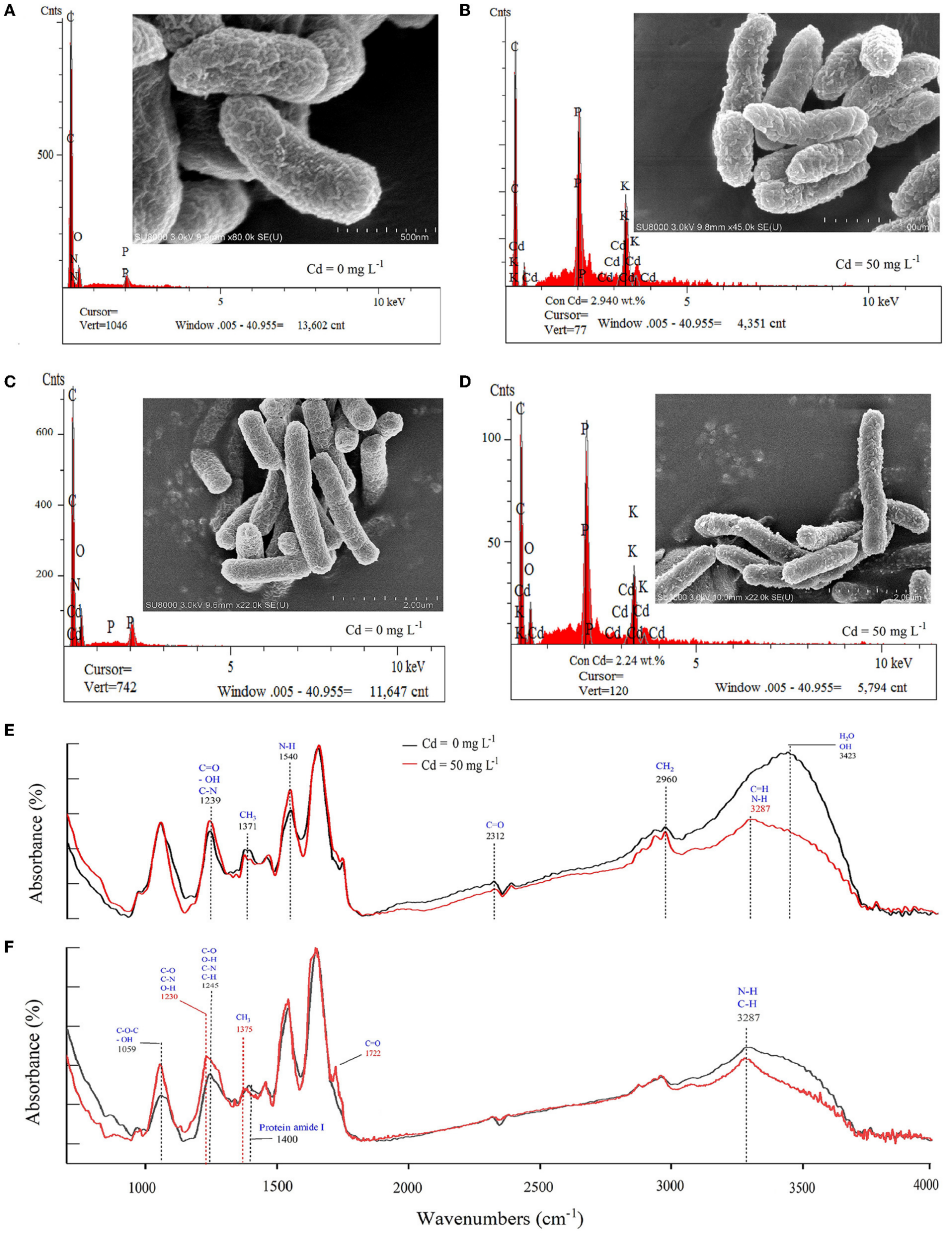
**(A–D)** Appearance and Cd elements on the cell surface of the two bacterial strains using SEM combined with the EDS analysis. **(A)** *Pseudomonas putida* 23483 without Cd solution. **(B)** *Pseudomonas putida* 23483 exposure to Cd solution (50 mg L^−1^) for 48 h. **(C)** *Bacillus sp*. GY16 without Cd solution. **(D)** *Bacillus sp*. GY16 exposure to Cd solution (50 mg L^−1^) for 48 h. **(E, F)** Functional groups on the cell surface of the microbial strains using the FTIR analysis. **(E)** *Pseudomonas putida* 23483. **(F)** *Bacillus sp*. GY16. The red lines represent strains without Cd solution, and the black lines represent strains exposed to Cd solution (50 mg L^−1^) for 48 h.

The SEM image and EDS analysis confirmed that Cd ions were attached to the cell surface (Figures 3A–D). The cell surface was smooth and the microbial cells were plumped without Cd stress (control) (Figures 3A, C), which was rough, atrophic, and with shiny bulky particles facing the stress of Cd of 50 mg L^−1^ (Figures 3B, D). This suggested that the damage to the cells and surface-active components were caused by Cd ions. The Cd ions exhibited in microbial cells (exposed to 50 mg L^−1^ of Cd) proved by the EDS atlas also supported the cell-sorption (Figure 3). The FTIR analysis showed changes in absorption peak areas of the cells exposed to 50 mg L^−1^ of Cd compared with that without Cd (Figures 3E, F), suggesting specific functional groups in the cell surface participated in cell-sorption (Pabst et al., 2010). For *P. putida*, the functional groups of C-H (CH_3_ symmetrical angle changed in 1371 cm^−1^, CH_2_ asymmetrical stretching in 2960 cm^−1^, and vibration as a move-left of 3423 to 3287 cm^−1^), C=O (such as stretching in 1239 and 2960 cm^−1^), N-H (such as stretching in 1540 cm^−1^, move-left of 3423 to 3287 cm^−1^), and O-H (stretching in 1239 and 3423 cm^−1^) (Zamil et al., 2008; Özdemir et al., 2009) were involved in biosorption of Cd (Figure 3E). For *Bacillus sp*. GY16, the functional groups of C-N, C-O, O-H (vibration as move-left in 1245 to 1230 cm^−1^, stretching in 1059 cm^−1^), C-H (vibration as move-left of protein amide I in 1400 to CH_3_ in 1375 cm^−1^, stretching in 3287 cm^−1^), and C=O (peaks in 1722 cm^−1^) (Figure 3F) were involved in biosorption of Cd (Zamil et al., 2008; Özdemir et al., 2009). Therefore, the number and type of these functional groups may determine the capacity and efficiency of biosorption (Figure 3), which could help reveal the chemical mechanism of bio-sorption of microorganisms.

### 3.4. Cd accumulation in rice plants and the chemical forms in soil affected by the strains

**Figure 4:**
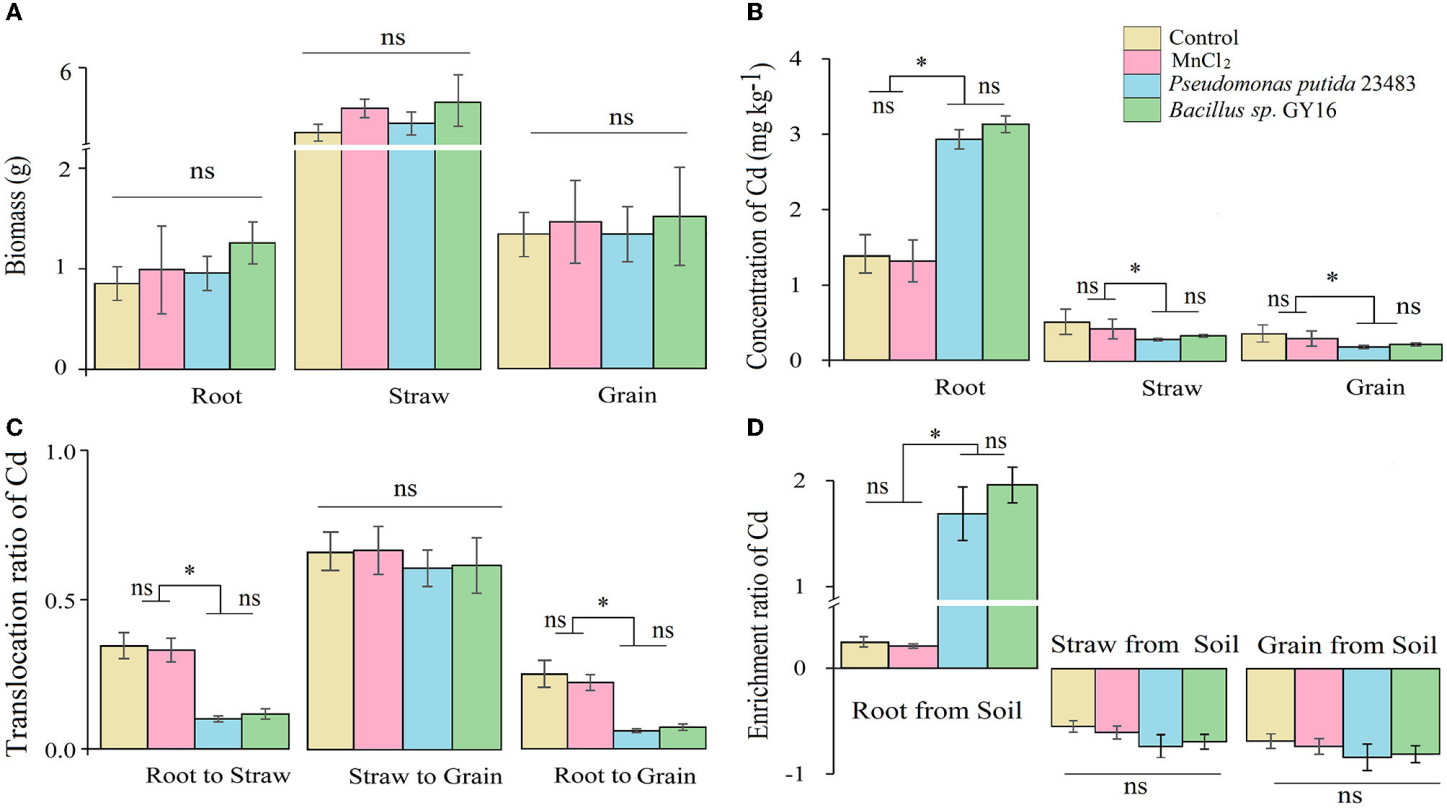
Biomass **(A)**, concentrations of Cd in root, straw, and grain **(B)**, the transmission ratio of Cd from root to straw and grain, **(C)** and enrichment ratio **(D)** without or with inoculation of microbial strains *Pseudomonas putida* 23483 and *Bacillus sp*. GY16. Bars with an asterisk (*) indicate significant differences among treatments determined by Tukey's HSD test at *P* < 0.05, and “ns” means not significant.

The root, straw, and grain biomass were neither affected (F= 15.856, *p* = 0.231) by the inoculation of *P. putida* and *Bacillus sp*. GY16 nor by the addition of MnCl_2_ (Figure 4A). This suggested that *P. putida* and *Bacillus sp*. GY16 accompanied by MnCl_2_ did not influence the growth and production of rice plants. Therefore, we suggested that the two Cd-resistant strains have no exceptional ability to promote the growth of the host plant in this concentration, not like other reported plant growth–promoting bacteria such as *Bradyrhizobium* sp. 750 (Dary et al., 2010), *Bacillus megaterium* H3, and *Neorhizobium huautlense* T1-17 (Li et al., 2017). Gratefully, Cd concentrations significantly decreased in rice straw and grain when inoculated with *P. putida* (*p* = 0.033, 0.014) and *Bacillus sp*. GY16 (*p* = 0.019, 0.030; Figure 4B), although Cd concentrations increased in roots when inoculated with *P. putida* (*p* = 0.016) and *Bacillus sp*. GY16 (*p* = 0.021; Figure 4B). This suggested microbial obstruction effect for Cd migration from soil to aboveground tissues. The reduced Cd uptake of rice in aboveground tissue may ascribe to IAA, siderophores, and ACC deaminase produced by bacteria evidenced by previous studies (Babu et al., 2015; Chen et al., 2016).

To reveal the effect of the two strains on the Cd accumulation in rice plants, we calculated the translocation ratio and the enrichment ratio along with the transport route of Cd in rice plants, such as from root to straw and to grain. The results showed that the enrichment ratio in root from soil (uptake ability) was significantly increased, whereas the translocation ratio of Cd from root to straw was significantly decreased when inoculated with *P. putida* (*p* = 0.018, 0.020) and *Bacillus sp*. GY16 (*p* = 0.011, 0.009) compared with the control (Figures 4C, D). This may be because microbes in the rhizosphere can develop a thick mucus barrier on the root tip and bind Cd ions inside microbial cell walls or on the root surface to limit its migration from root to plant aboveground tissues (Pál et al., 2006). As a result, the translocation ratio of Cd from root to grain was decreased when inoculated with *P. putida* (*p* = 0.034) and *Bacillus sp*. GY16 (*p* = 0.042) (Figure 4C), and the translocation ratio of Cd from rice straw to grain was not significantly different among the groups because of root binding (Figure 4C). This suggested that the inoculation of *P. putida* and *Bacillus sp*. GY16 allows soil Cd ions to mainly enrich and fix in plant roots and reduce their translocation in straw and grain from the root. Therefore, the microbial inhibition of Cd in rice mainly occurred in the root and was achieved by preventing Cd transmission and enrichment in the straw and grain from the soil.

**Figure 5:**
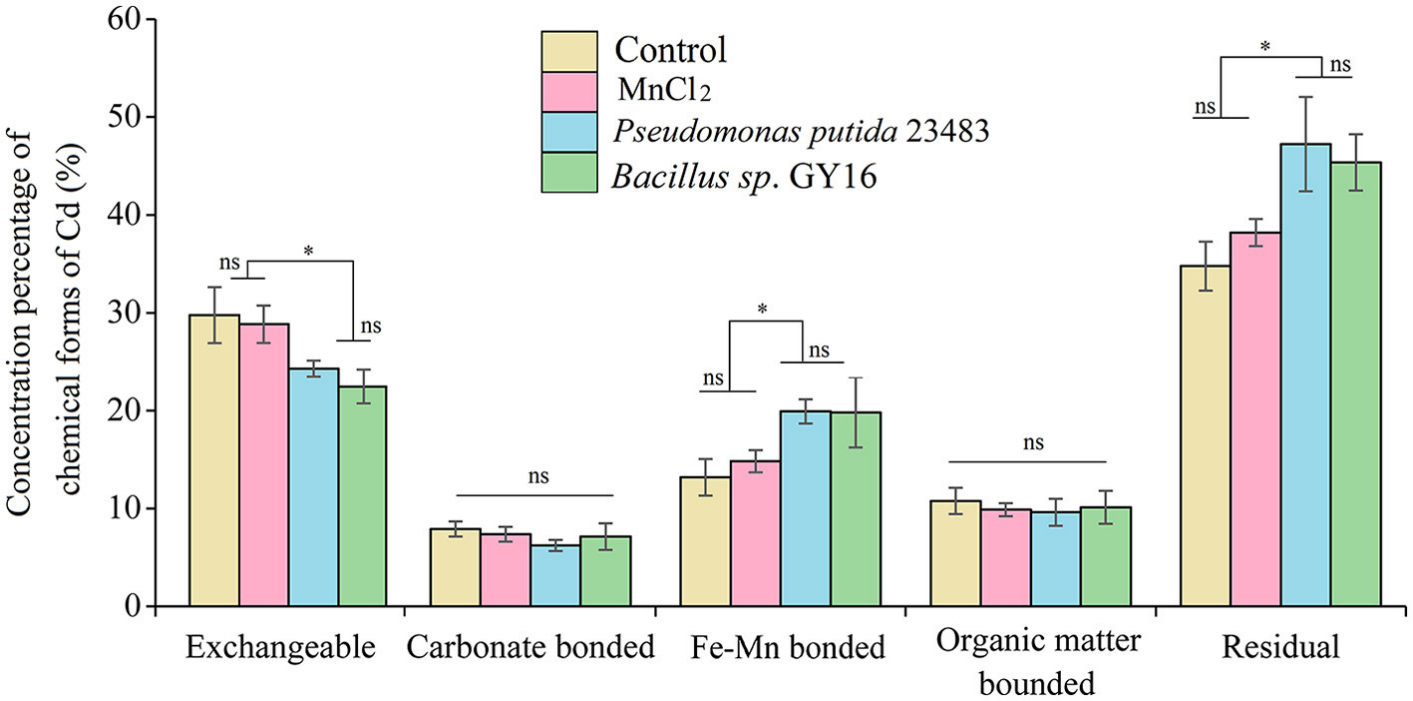
Fractionation of Cd in soil without or with inoculation of microbial strains *Pseudomonas putida* 23483 and *Bacillus sp*. GY16. Bars with an asterisk (*) indicate significant differences among treatments determined by Tukey's HSD test at *P* < 0.05, and “ns” means not significant.

Furthermore, the immobilization of Cd in soil by functional microbes is crucial to reduce its uptake by plants (Kuffner et al., 2010; Dourado et al., 2013; Ahmad et al., 2014). The transformation of chemical forms of soil Cd could directly affect its accumulation in rice plants because only the exchangeable Cd in the soil can be uptaken by plants. Our results showed that the external addition of MnCl_2_ of this amount in soil did not affect the chemical forms of Cd, but the concentration of soil exchangeable Cd was significantly decreased when inoculated with *P. putida* (*p* = 0.007) and *Bacillus sp*. GY16 (*p* = 0.004) accompanied by MnCl_2_ addition (Figure 5). This was consistent with a previous study that suggested that resistant bacteria reduced Cd accumulation in grains of rice by reducing the available Cd in the soil (Lin et al., 2016). Notably, the inoculation of *P. putida* and *Bacillus sp*. GY16 accompanied by MnCl_2_ significantly increased the Cd concentrations of the Fe–Mn binding form (*p*_*P*.*putida*_ = 0.018; *p*_GY16_ = 0.023) and the residual form (*p*_*P*.*putida*_ = 0.034; *p*_GY16_ = 0.028) compared with the control (Figure 5). This suggested that the strains play important roles in preventing soil Cd from converting to a soluble form but existing as combined forms such as iron–manganese oxide binding form and residue form, ascribed to its characteristics of manganese-oxidizing (He et al., 2008). It has been demonstrated that microorganisms can speed up the rate of Mn oxidization up to 10^5^ times faster than abiotic Mn oxidization. We inoculated the strains accompanied by MnCl_2_ in soil because the process of Mn bio-oxidization needs a supplement of active Mn^2+^, but most of the Mn^2+^ in the soil exists in the form of compounds (Kim et al., 2003; Tebo et al., 2004). However, the concentrations of carbonate binding and organic binding forms of Cd were not significantly different among treatments (Figure 5). Nevertheless, the interaction mechanism between Cd-contaminated soil, rice plants, and the functional microbes needs further study.

## 4. Conclusion

**Figure 6:**
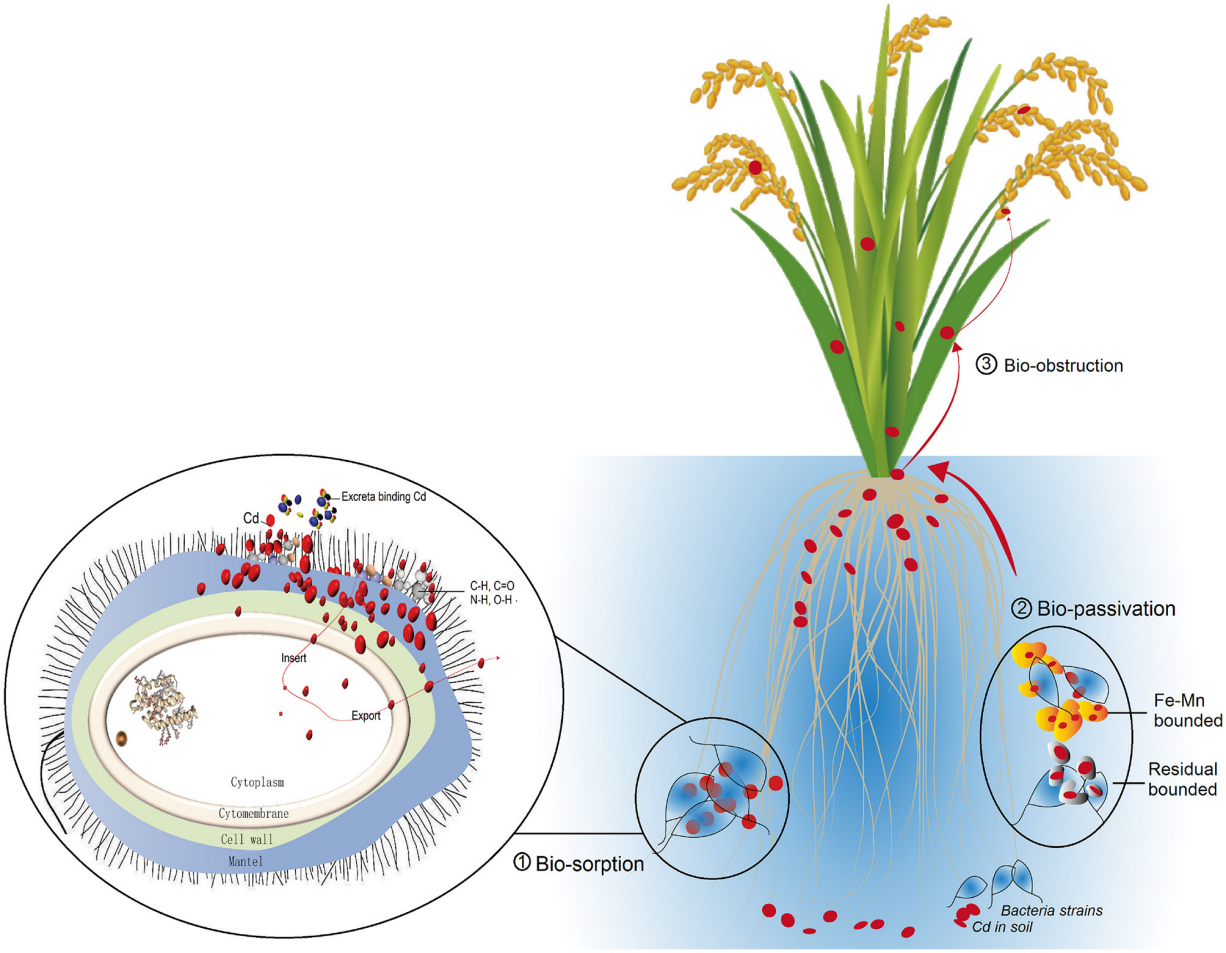
Conceptual graph summarizes the Cd dispose mechanism of the two microbial strains, including biosorption mainly *via* cell mantel and cell wall (Bio-sorption), bio-passivation of soil Cd as Fe–Mn combined form (Bio-passivation), and the bio-obstruction (Bio-obstruction) achieved by transmission from root to straw and grains.

Our study revealed that Cd ions could be efficiently removed by *P. putida* and *Bacillus sp*. GY16 through biosorption of the cell wall. The two strains do not significantly affect the rice plant growth and production but have microbial obstruction effect on Cd enrichment from soil to grain *via* root binding of bioactive Cd and soil passivation as Fe–Mn compound and residual combination forms (Figure 6). Therefore, we advocate the application of those high Cd-resistant strains for Cd pollution remediation in soil or water as an efficient and environmentally friendly approach and to prove the bioremediation consequence in field conditions. Furthermore, we advocate some microbial composite eco-materials such as biological Mn oxide to be fabricated to improve the biosorption efficiency in complicated soil or water conditions.

### 4.1. Environmental implication

The two high-tolerant microbial strains can efficiently remove Cd ions and play an important role in preventing soil Cd from converting to bioactive form but passivating to Fe–Mn combined form, eventually decreasing the accumulation of Cd on rice grain, and achieving bio-obstruction of Cd from soil to rice grain. We advocate that those high Cd-resistant strains be used in soil or water remediation of Cd pollution in an efficient and eco-friendly way, and some microbial composite eco-materials such as biological Mn oxide be fabricated to improve the biosorption efficiency in complicated soil or water conditions.

## Data availability statement

The original contributions presented in the study are included in the article/supplementary material.

## Author contributions

JL, H-YC, and J-ZH conceived and designed the experiments. JL, Y-KG, and C-QL performed the experiments, analyzed the data, prepared figures and/or tables, and wrote the manuscript. QZ helped to determine the samples. All authors approved the final draft.
